# Molecular epidemiology and evolutionary genetics of *Mycobacterium tuberculosis *in Taipei

**DOI:** 10.1186/1471-2334-8-170

**Published:** 2008-12-22

**Authors:** Horng-Yunn Dou, Fan-Chen Tseng, Chih-Wei Lin, Jia-Ru Chang, Jun-Ren Sun, Wen-Shing Tsai, Shi-Yi Lee, Ih-Jen Su, Jang-Jih Lu

**Affiliations:** 1 Division of Clinical Research, National Health Research Institutes, Zhunan, Taiwan. 35 Keyan Road, Zhunan, Miaoli County 350, Taiwan, Republic of China; 2 Division of Clinical Pathology, Department of Pathology, Tri-Service General Hospital and National Defense Medical Center, 325 Sec. 2 Chenggong Rd., Taipei 114, Taiwan, Republic of China; 3 Department of Laboratory Medicine, China Medical University Hospital, 2, Yuh-Der Road, Taichung 404, Taiwan, Republic of China

## Abstract

**Background:**

The control of tuberculosis in densely populated cities is complicated by close human-to-human contacts and potential transmission of pathogens from multiple sources. We conducted a molecular epidemiologic analysis of 356 *Mycobacterium tuberculosis *(MTB) isolates from patients presenting pulmonary tuberculosis in metropolitan Taipei. Classical antibiogram studies and genetic characterization, using mycobacterial interspersed repetitive-unit-variable-number tandem-repeat (MIRU-VNTR) typing and spoligotyping, were applied after culture.

**Methods:**

A total of 356 isolates were genotyped by standard spoligotyping and the strains were compared with in the international spoligotyping database (SpolDB4). All isolates were also categorized using the 15 loci MIRU-VNTR typing method and combin with *NTF *locus and RD deletion analyses.

**Results:**

Of 356 isolates spoligotyped, 290 (81.4%) displayed known spoligotypes and 66 were not identified in the database. Major spoligotypes found were Beijing lineages (52.5%), followed by Haarlem lineages (13.5%) and EAI plus EAI-like lineages (11%). When MIRU-VNTR was employed, 140 patterns were identified, including 36 clusters by 252 isolates and 104 unique patterns, and the largest cluster comprised 95 isolates from the Beijing family. The combination of spoligotyping and MIRU-VNTR revealed that 236 (67%) of the 356 isolates were clustered in 43 genotypes. Strains of the Beijing family was more likely to be of modern strain and a higher percentage of multiple drug resistance than other families combined (P = 0.08). Patients infected with Beijing strains were younger than those with other strains (mean 58.7 vs. 64.2, p = 0.02). Moreover, 85.3% of infected persons younger than 25 years had Beijing modern strain, suggesting a possible recent spread in the young population by this family of TB strain in Taipei.

**Conclusion:**

Our data on MTB genotype in Taipei suggest that MTB infection has not been optimally controlled. Control efforts should be reinforced in view of the high prevalence of the Beijing strain in young population and association with drug resistance.

## Background

Tuberculosis (TB) remains a worldwide healthcare concern and has been characterized as an epidemic by World Health Organization (WHO). It is estimated one third of the world's population has been infected with *Mycobacterium tuberculosis *(MTB) and that 3 million people will die of the disease per year between now and 2010. The distribution of TB in different geographic regions is characterized by the prevalence of different MTB strains with varied virulence and drug resistance. Both environmental and host factors are responsible for the transmission and prevalence of different MTB strains. Although both the incidence and mortality rates of TB in Taiwan have shown a steady decline since 1950, TB remains a leading notifiable infectious disease on the island. In 2001, 14,486 cases were reported, with a notification rate of 64.9 per 100,000 people.

At the molecular level, the global TB epidemic consists of multiple genotype-specific subepidemics. Different MTB genotypes can be identified by variation in certain well-characterized repetitive sequences, such as the IS*6110 *transposable element and the direct repeat region [2]. The Beijing genotype family is well recognized as having a distinct genetic signature, and it is genetically highly conserved [3] even though sequence polymorphisms have identified four monophyletic subgroups [4]. It is dispersed worldwide yet predominates in certain geographic areas, particularly in parts of Asia [5,6] and Russia [7]. Its prevalence in the patient populations of recent studies in Vietnam and Russia suggests the recent spread to those areas [8]. It has been proposed that "Beijing" should be regarded as an emerging genotype family [9].

The association between drug resistance and the Beijing genotype is well documented in recent medical literature [3,4,6,10-13]. The geographic variability observed in this association [4], along with the frequent clustering of resistant genotypes and their successful spread within the Russian prison system [7,13] suggests recent colonial expansion. This is further supported by the evidence that some strains of the Beijing genotype family retain fitness despite the acquisition of drug resistance [13]. The Haarlem family genotype has a similar relationship with drug resistance and rapid clonal expansion [14]. The association of these genotype families with drug-resistant outbreaks clearly demonstrates their epidemic potential [14,15]. From a TB-control point of view, it is relevant to understand whether specific genotype families are overrepresented among drug-resistant cases and, in particular, if these resistant strains are successfully transmitted within the community. Taipei is a metropolitan city in northern Taiwan with a population of 2.3 million inhabiting a basin of 272 square km. The population of Taipei includes Han Chinese whose ancestors migrated to this island in the 16th century, the veterans who retreated to the island in the late 1940s during the Chinese civil war, and the Taiwanese Aborigines, who have resided on this island since before the 16th century [16]. The prevalence of TB in large urban areas is complicated by the close human-to-human contacts and potential multiple sources of MTB strains from different ethnic and migratory populations. The goals of this study were therefore to characterize the prevalence of genotypes, cluster pattern, and drug resistance of MTB isolates in Taipei to provide information for potential transmission and formulation of effective infection-control policy.

## Methods

### Mycobacterial strains and genomic DNA

A total of 356 samples were randomly collected between 2002 and 2004 from 356 patients at the Tri-Service General Hospital, a large medical center that handles a substantial number of TB patients referred from hospitals throughout Taipei. All of the patients were sputum microscopy positive and culture positive. Mycobacterial genomic DNA was extracted from cultured cells as described previously [17,18]. Resuspending mycobacterial colonies in 100 to 200 μl of distilled H_2_O and incubating them at 85°C for 30 min obtained genomic DNA. After centrifugation of the suspension, the supernatant containing the DNA was removed and stored at -20°C until further use. The study protocol has been approved by the institutional review board of the National Health Research Institutes, Taiwan.

### Spoligotyping and spoligotype analysis

Spoligotyping was carried out according to the manufacturer's instructions (Isogen Bioscience B.V., Maarsen, Netherlands). The resulting spoligotypes were documented under a binary code representing either a positive or negative hybridization result (n and o, respectively) and analyzed using the Excel program for grouping and ordering of the patterns. Spoligotypes common to more than one strain were designated as shared types (ST) and assigned a shared international type number (SIT) according to the updated version of the international spoligotype database SpolDB4 [19].

### PCR and MIRU analysis

PCRs were carried out using the PCR reagent system (Gibco-BRL). Sequences of primers used for amplification of 12 MIRU loci and 3 ETR loci (A, B, C) were selected according to descriptions in other studies [20]. Five microliters from fivefold-diluted DNA solutions were added to a final volume of 50 μl containing 0.2 μl of DNA polymerase (1 U); 0.2 mM each of dATP, dCTP, dGTP, and dTTP; 5 μl of PCR buffer; 0.4 μM (2 μM for locus 7) of primers; and 1 to 3.5 mM of MgCl_2_. The primers and MgCl_2 _concentrations used were as described by Mazars et al. [21]. The PCR fragments were analyzed by agarose gel electrophoresis with 1.5% agarose. The sizes of the amplicons were estimated by comparison with 50- and 100-bp ladders. The MIRU copy number per locus was calculated by using the conventions described by Supply et al. [22].

### TbD1 Analysis

According to Brosch et al. [23], TbD1 is specifically present in the ancestral lineage of MTB. The presence of TbD1 was analyzed by PCR. Briefly, two PCR assays were performed per isolate by using either primers complementary to the sequences flanking the deleted region or primers complementary to the internal sequences. For the isolates that did (TbD1+) or did not (TbD1-) contain the TbD1 region, an amplicon was obtained only with internal primers or only with flanking primers, respectively.

### *NTF *locus and RD deletion analysis

A multiplex PCR approach was used to determine possible IS*6110 *insertion(s) in the *NTF *region of *M. tuberculosis *strains. The method, including primers within the *NTF *region as well as the IS6110 sequence and PCR parameters, was adapted from a previously described paper by Plikaytis et al. [24].

A primer set was used to check for the presence or absence of RD105, RD181, RD150, RD142, and RD207. The PCR mixture consisted of 0.2 μg DNA template, 13.9 μl Q buffer, 5 μl 5× buffer, 4 μl 10 mM deoxynucleoside triphosphates, 1 μl of each primer (10 pmol/μl), 1μl DMSO, and 0.6 μl Herculase II Fusion DNA polymerase (STRATAGENE, USA). Sterile water was used to dilute the mixture up to 25 μl. A detailed explanation of this methodology has been described [25-27].

### Drug resistance testing

The proportional method for drug susceptibility testing (DST) of MTB was performed as described previously [28]. Briefly, for each drug a 1:10 dilution of standardized suspensions was inoculated onto the control and drug-containing media. The extent of growth in the absence or presence of the drug was compared and expressed as a percentage. If growth at the critical concentration of a drug was >1%, the isolate was considered to be clinically resistant. 7H10 agar with 0.2 or 1 mg/l isoniazid (INH), 1 or 5 mg/l rifampicin, 5 or 10 mg/l ethambutol, and 5 or 10 mg/l streptomycin was used.

### Statistical analyses

Frequencies of multiple-drug resistance (MDR) among different genotype families based on spoligotyping were compared with a chi-square test, or a Fisher's exact test when any of the cells had expected counts ≤ 5. The extent of association was expressed as an odds ratio (OR) and 95% confidence interval (95% C.I.). All statistical tests were two-sided; and statistical significance was set at a p-value <0.05.

Patients in this study can be classified into two groups, characterized by clustered and non-clustered MTB isolates. A possible cluster is defined as two or more patients' strains with identical genetic patterns defined by the MIRU-VNTR typing; patients' strains with unmatched genetic profiles were considered non-clustered. Previous literatures have suggested that clusters may be assumed to have arisen from recent transmission; and the clustering rate was used to determine the amount of recent transmission in this population [29,30]. The patients' strains with the same genetic pattern may represent an epidemiologically linked cluster. Therefore, the minimum estimate of the proportion of *M. tuberculosis *cases related to recent transmission can be calculated as (number of clustered patients minus the number of clusters)/total number of patients (Additional file 1).

## Results

### Analysis of the spoligotyping patterns

**Figure 1:**
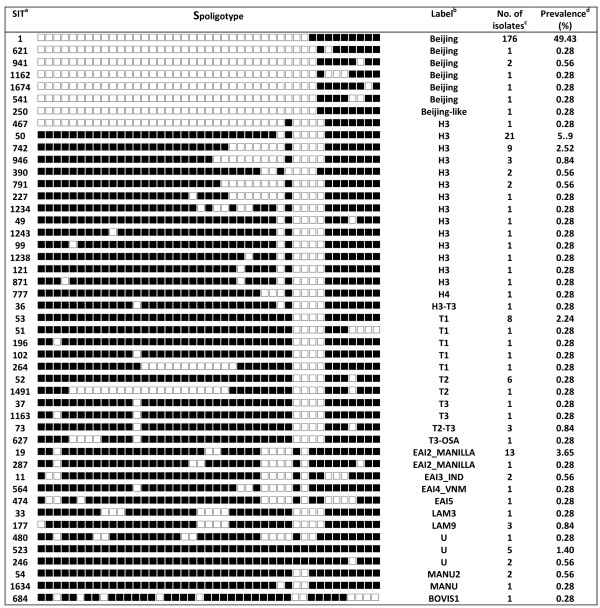
**Spoligotypes of 290 isolates with a shared international type (SIT) number in SpolDB4**. ^a ^Shared international type (SIT), international spoligotype database SpolDB4 . ^b ^Label representing spoligotype families as assigned in the international spoligotype database SpolDB4. ^c ^Number of isolates in this study. ^d ^Prevalence, representing the number of isolates with a common SIT relative to the total number of isolates from the same database (356) classified by SIT from Tri-Service General Hospital (expressed as a percentile).

**Figure 2:**
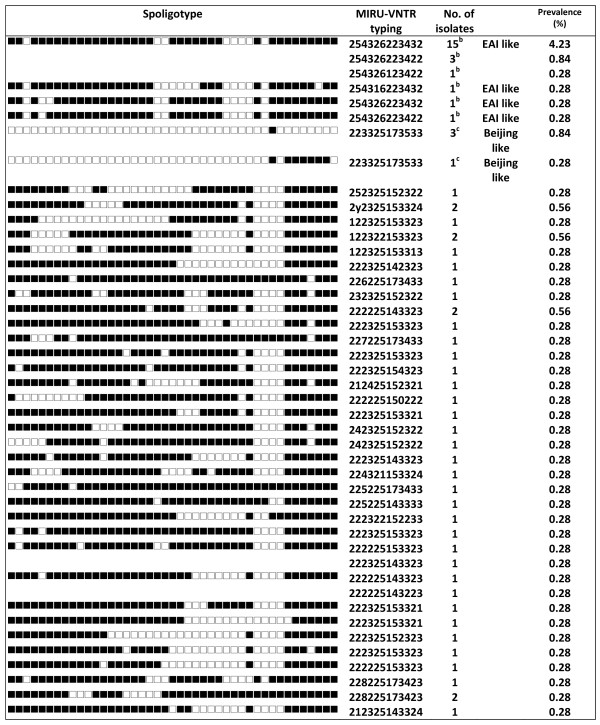
**Spoligotypes of 66 orphan strains and clusters of spoligotypes not identified in SpolDB4 by a SIT number^a^**. ^a ^Shared international type (SIT), international spoligotype database SpolDB4 . ^b ^TBD1 positive, ^c ^RD105 deletion.

**Figure 3:**
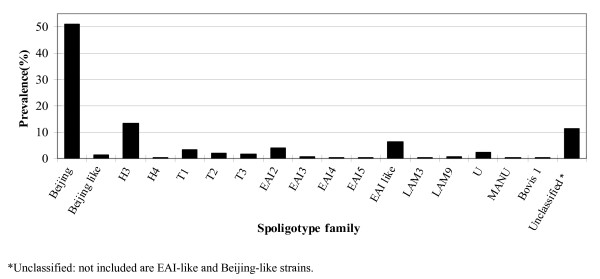
**Prevalence of the major spoligotype families and subfamilies of 356 *M. tuberculosis *isolates**.

During the study period, 356 patients were diagnosed with culture-confirmed TB. Molecular analysis showed that all TB cases were caused by *M. tuberculosis*, except one by *Mycobacterium bovis*. The median age of these patient was 61.2 years, and 70%(251/356) were male. Spoligotyping and drug susceptibility testing (DST) were performed on all of the specimens. Of the 356 isolates analyzed, spoligotypes from 290 isolates (81.4%) were classified according to SpolDB4 into one of the 47 shared international types (SITs) (Figure 1). Of the remaining 66 isolates, 40 patterns were not identified in the database, 22 were of the East African-India (EAI), 4 were of the Beijing, and the other 40 (11.2%) were orphans (Figure 2). Of the 47 defined spoligotypes, the most frequent strain found was the Beijing spoligotype ST1 (49.43%), followed by ST50 (5.9%) of the Haarlem strains, and ST19 (3.65%) of the EAI_2 Manilla strains (Figure 1). The Beijing family was the most prevalent genotype, identified in 187/356 (52.5%) isolates, followed by the Haarlem family, identified in 48/356 (13.5%) isolates (Table 1). In these novel spoligotypes, 22 strains were found to be TBD1 positive and are further characterized by the absence of DR spacers 29 to 32 and 34 and the presence of spacer 33. Based on this result, these new spoligotypes belong to the East African-India (EAI) family. There are two novel spoligotypes with the RD 105 deletion, indicating their membership in the Beijing family (Figure 2). Of all the isolates studied, the most prevalent subfamilies after the Beijing family (52.5%) were H3 (13.2%) and EAI-like (6.2%) (Figure 3). Among all of the isolates, 63 (18%) displayed unique spoligotypes, and 293 (82%) displayed one of 24 spoligotypes.

**Table 1:** Mycobacterial genotype and drug resistance in patients with culture-confirmed tuberculosis

		**No. of isolates with DST result**		
		
**Genotype family**	**No. of isolates (%)**	**MDR(%)**	**Any one drug(%)**	**All sensitivity(%)**
Beijing^a^	187(52.5)	8(4.2)	36(19.4)	143(76.4)
Haarlem	48(13.5)	0	9(18.8)	39(81.2)
EAI^b^	40(11.2)	0	3(7.5)	37(92.5)
T	25(7.1)	0	8(32.0)	17(68.0)
'Others'^c^(LAM, U, MANU, Bovis1)	16(4.5)	0	1(6.3)	15(93.7)
Unclassified^d^	40(11.2)	2(5)	8(20)	30(75)

Total	356	10(2.8)	65(18.2)	281(79)

^a ^Including Beijing-like strains;

^b ^Including EA-like strains;

^c^'Others', all genotype families with a frequency of less than 10 cases;

^d ^unclassified, no internationally recognized genotype family assigned, based on the SpolDB4 spoligotype database.

The age distribution of patients in different genotype, which include Beijing, Haarlem, EAT, T and others genotype of average age in year respectively is 58.7, 62.1, 66.9, 61.4, 65.3. Statistical analyses by T-test demonstrated that patient infects with the Beijing family (mean = 58.7) were statistically younger than those infected with other geno-families (mean = 64.2) with a p value of 0.02. Moreover, 68.5% of patients 30 year-old or younger were infected with Beijing strains as compared to 50% of those older than 75 with Beijing strains, giving an odds ratio of 2.18 with 95% C.I. = 1.11–4.28 and p = 0.02 (Table 2). The association between young age and Beijing strains was even stronger in younger ages: 85.3% of people 25 year-old or less had Beijing strain, and when compared with those more than 75 year-old produced an odds ratio of 5.8 with p = 0.0002 (Table 2). These results indicated that young population in Taipei were more likely to be infected with Beijing strains than with other strains and as compared to older age groups, and thus suggested a possible recent spread of the Beijing genotype in the young generation in this area.

**Table 2:** Beijing family genotype *M. tuberculosis *isolates from 356 tuberculosis cases

	**No.(%) isolates No**.	**(%) of Beijing isolates**	**Odds Ratio**	**95% C.I**.	**p-value**
**Age group (yr)**	**356**	**187 (52.53)**			
≦ 25	34 (9.55)	29 (85.29)	5.80	2.11–15.98	0.0002
≦ 30	54 (15.17)	37 (68.52)	2.18	1.11–4.28	0.02

31–60	95 (26.69)	50 (52.63)	1.11	0.65–1.90	0.7

61–75	85 (23.88)	39 (45.88)	0.85	0.49–1.48	0.56

≧ 76	122 (34.27)	61 (50.00)	1	reference group	

### Typing of strains and clustering analysis by MIRU-VNTR

All 356 isolates were also categorized using the MIRU-VNTR typing method, which detected 140 different patterns, comprising 36 clusters formed by 252 isolates and 104 unique patterns formed by 104 isolates (Table 3). The largest cluster comprised 95 from the Beijing family, with an MIRU-VNTR profile of 223325173533. Of 187 Beijing strains as defined by spoligotyping, MIRU-VNTR typing further divided them into 47 different patterns, comprising 15 clusters formed by 155 isolates and 32 unique patterns formed by 32 isolates. Of 48 Harrlem strains, 24 different MIRU-VNTR patterns were found, which consisted of 5 clusters and 19 unique MIRU-VNTR genotypes. The largest cluster comprised 10 in the Haarlem family, with an MIRU profile of 222225153323. The T family was divided into 19 different MIRU genotypes.

**Table 3:** Degree of discrimination obtained with two typing methods individually and combined

**Method**	**No. of different patterns**	**No. of unique isolates**	**No. of clustered isolates**	**No. of clusters**
Spoligotyping alone	87	63(18%)	293(82%)	24
MIRU-VNTR typing alone	140	104(29%)	252(71%)	36
Spoligotyping+ MIRU-VNTR typing	181	120(33%)	236(67%)	43

A test population of 356 MTB isolates classified by spoligotyping analysis into six MTB lineages was typed using 15-locus (including ETR-A, B, C) MIRU-VNTR profiling. The combination of spoligotyping and MIRU-VNTR typing for the 356 isolates revealed that 120 (33%) have unique genotypes and 236 (67%) can be grouped into one of 43 genotypes. The minimum estimate for the proportion of TB in the study population due to recent transmission is estimated around 54% ([236–43]/356) (Table 3).

### *NTF *locus and RD deletion analysis

**Figure 4:**
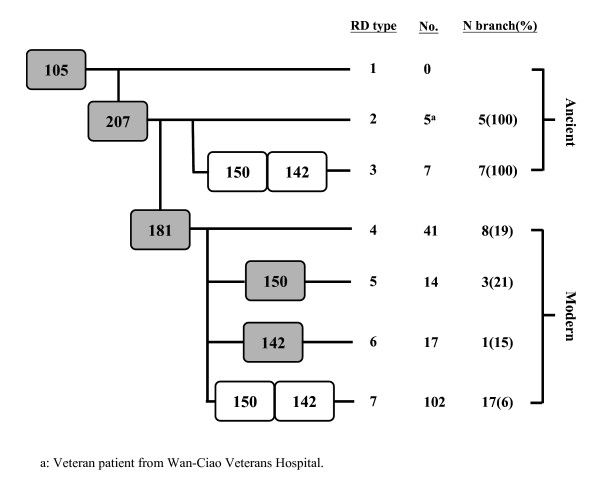
**Beijing family subgroup structure**. Scheme of the proposed evolutionary pathway of the Beijing lineages, based on the deletion of genomic regions (RD, region of difference) showed inrectangles and on the NTF region with IS6110 insertions. The gray rectangles are lineages that have been reported previously, while the white rectangles represent proposed new lineages that are identified in the current study study.

The RD105 (regions of difference) LSP appears in all Beijing strains examined to date and can be used to define the set of strains belonging to this lineage. Further subdivisions of the *M. tuberculosis *Beijing lineage are made on the basis of the variable appearance of the RD207, RD181, RD150, and RD142 deletions. In the present study, we refer to group 1 Beijing strains as those that contain only the RD105 deletion, while group 2 to 7 Beijing strains also contain RD105 and RD207 deletion (group 2); RD105, RD207, RD142, and RD150 deletion (group 3); RD105, RD207, and RD181 deletion (group 4); RD105, RD207, RD181, and RD150 deletion (group 5); RD105, RD207, RD181, and RD142 deletion (group 6); and RD105, RD207, RD181, RD150, and RD142 deletion (group 7). Strains in groups 1–3 have neither deletion of RD181 nor insertion of IS6110 in NTF region, and can be thought of as being "ancient" to the "modern" Beijing lineage (Figure 4). Results of RD analyses suggest that most of Beijing isolates in general population were modern strains (96%, 174/181).

### Drug resistance patterns

Of the 356 strains in this study, 281 isolates (79%) were sensitive to all four of the first-line agents tested, and 75 were resistant to at least one drug, 2.8% are multidrug resistant (MDR) (Table 1). Analysis of the association between MDR and genotypes (as determined by spoligotyping) showed that the Beijing genotype is more likely to be MDR than all other genotypes (Haarlem, T, EAI, others, and orphan combined) [p = 0.08, OR = 3.73, and 95% C.I. = (0.78–17.83)]. The EAI family is significantly more likely to be sensitive to all drugs than are other genotypes [p = 0.02, OR = 3.64, and 95% C.I. = (1.09–12.15)]. Interestingly among the orphan strains, 5% were MDR and 20% were resistant to one drug, showing a distribution similar to that of the Beijing family.

## Discussion

Tuberculosis remains a major public health issue in Taiwan and throughout the world. Recent advances in molecular technology such as spoligotyping and MIRU-VNTR typing provide a powerful tool to analyze MTB genotype and transmission patterns, which should be valuable for development of effective infection-control policy.

Among the 356 samples we analyzed in our study, 65% isolates would be considered to be potentially clustered based on 15 loci MIRU-VNTR typing [29]. Such rate is higher than that reported for other major cities such as San Francisco or New York, but similar to that from the Netherlands and Denmark, where the average of recent transmission rate ranged between 35% and 45% [30-33]. In densely populated areas, such as Northern Malawi, South African, clustering could be even higher, approaching 70% in some reports [34-36]. However, the rate of clustering in this study may have been overestimated due to the insufficient number of MIRU-VNTR loci we used, especially in an ares with high prevalence of Beijing strains[37]. Typing by additional loci, such as the 24 loci MIRU, is required to better differentiate the genetic relationship, clustering and possible transmission chain of all Beijing strains. Since typing methods of 24 loci MIRU were published in 2006, we attempted to set up the experiment protocol in our laboratory for the other 8 loci on top of the 16 loci we had used (the 15 shown in this paper plus the "MPTR-A"). Typing for 6 of the 8 loci was successfully established and performed on 52 Beijing strains that were from 15 clusters based on the 15 loci MIRU typing in this study. Our preliminary data using the additional 6 loci revealed that the same clustering patterns as that by the 16 loci were observed in 50% of the 52 Beijing strains analyzed. Therefore, the addition of the new VNTR loci to MIRU analysis is required to clarify the clustering and transmission issue in Taipei city in the future.

The modern MTB strains such as the Beijing, Haarlem, EAI, and T clusters comprise the causal agents of major epidemics. This study revealed the Beijing strain as the dominant pathogen for up to 52.5% of cases in Taipei, similar to the level in a recent report for Taiwan as a whole [38]. The majority of MTB strains of the Beijing family originated from the area in and around Beijing, China, and strains of this family were found to be dominant in neighboring countries such as Indonesia (44%), South Korea (72%), Thailand (44%), and Vietnam (53%) [3,5,8]. In contrast to the predominance of the Beijing genotype in many Asian countries, a low frequency (3%) of this genotype was reported among the strains in India [39]. Strains of the Beijing family have also been found in Europe, Africa, and the United States. The W strain, which caused a large outbreak of multidrug-resistant TB in New York and other U.S. cities, also belongs to the Beijing family [40]. In countries neighboring Taiwan, rates of infection with the Beijing family strains are higher than those in more distant countries, suggesting that the Beijing family may have radiated from the Beijing area to other regions. Based on the epidemiologic data, the Beijing strains appear to have a growth advantage over other strains, enabling them to circulate better in the population [3,5,8]. Moreover, we also demonstrated that Beijing family strains were associated with MDR phenotypes in this study (p = 0.08), a finding similar to that in the recent report from Taiwan [38]. Association between Beijing strains and MDR varies worldwide. Although such an association was reported in studies in the United States, Estonia, and Vietnam [41], it has not been noted in countries such as China and Indonesia, where representation of Beijing strains in the population is higher [42].

A total of 187 strains in the Beijing cluster identified by spoligotyping were further discriminated by MIRU-VNTR analysis. A total of 155 of the 187 strains were clustered into 15 groups, each consisting of 2 to 95 strains; and remaining 32 strains were found to have unique patterns. This study further showed that MTB isolates grouped into the Beijing family by spoligotyping have a similar grouping pattern when other genetic markers such as MIRU-VNTR typing are used. This was borne out by the fact that all MIRU-VNTR patterns of Beijing family strains were highly similar, differing only in copy numbers for one to three loci. In this study, all isolates that contain ≥ 2 repeats in the MIRU-VNTR locus 24 belong to the ancestral (TbD1+) group; and all but 1 isolate containing two repeat units in locus 24 belong to the modern (TbD1-) groups. In contrast, we found that ST480 of the U lineage has the MIRU-VNTR profile 254326223432 and TBD1+. The genetic characteristics of ST480 are very similar to those of the EAI family. Frequencies of TbD1+/EAI isolates have recently been reported to range from 25% to 50% in Bangladesh [43,44] and Singapore [45]. A frequency of 8% has been reported in a study that used spoligotyping alone for genetic characterization of 105 isolates from the Delhi area [46]. Our analysis found that 5.5% of the samples were TbD1+/EAI isolates, while another 5.5% were TbD1+/new EAI spoligotype. The Haarlem isolates accounted for 13.5% in this study. Preliminary studies on the MTB strain distribution in eastern Taiwan's Hualien County, where Taiwanese Aborigines comprise a relatively large percentage of the population, showed a predominance of the Haarlem strain of up to 45% [47]. Since Taiwan was colonized by the Dutch in the 17th century, it is conceivable that the Haarlem strain is dominant in Taiwanese Aborigines. In addition to the Beijing and Haarlem strains, we identified the EAI family, the T family, the Latin American-Mediterranean family (LAM), the U family, and the MANU family of MTB in our population in Taipei. In addition to the identified predominant groups, we were able to identify the occurrence of rare clusters or localized STs listed in SpolDB4.0 that had previously been found in America, Australia, and Europe, with more found in countries neighboring Taiwan, such as Vietnam, Malaysia, the Philippines, and China.

TB occurs partly as a primary disease (typically defined as occurring within 5 years of infection) and partly as an endogenous reactivation or exogenous reinfection (occurring >5 years after infection) [48]. With increasing age, a decreasing proportion of cases are due to primary TB. Thus, the association of the Beijing genotype and young age suggests a recent spread of the Beijing genotype in Taipei. Anh et al. [49] reported that *M. tuberculosis *isolates of the Beijing genotype was less associated with BCG vaccination but was frequently associated with younger age in Vietnam. Lopez et al [50] used the mouse model of pulmonary tuberculosis to investigate the protective efficacy of BCG against these different strains and found that BCG was least protective against the Beijing strain. In contrast, Chan et al. did not find in Hong Kong any association between the Beijing genotype and younger age but did find a weak association with isoniazid (INH) resistance [51]. Although Taiwan executes comprehensively the BCG vaccination for more 40 years, the predominance of Beijing family strain in young population (85%, below 25 years of age) in this study suggest that BCG may fail to protect adequately the young people infected with the Beijing strain MTB.

The Beijing family can be further grouped into ancestral, modern, and recent strains by NTF locus analysis [8] and RD deletion analysis [4,52], suggesting the strains' temporal evolution or transmission in migratory populations [16]. According to our previous study, the distribution of the Beijing sub-lineage with intact NTF region (ancient) was 19% in the general population, 24% in the veterans, and 50% in Aborigines in Taiwan [47]. We speculate the group 3 needed for 500 years from the evolution by group 1, group 4 to group 7 only need for 50 years, obviously modern strain genome was unstable and perhaps this instability was conducive to its fast spread. According to the view of evolution, existence of RD181 region or not, boundary to become modern and ancient lineage, infer RD181 perhaps (contain Rv2262c, Rv2263) the gene included may relate to the maintenance of genome stability. The hypothetical protein RV2262c may involve in protein modification and repair, and the hypothetical protein RV2263 involve in oxidoreduction . The genome instability may be caused by these gene deletions.

## Conclusion

This study gives a first overview of the *M. tuberculosis *strains circulating in metropolitan Taipei. Based on a combination of spoligotyping and MIRU-VNTR, our preliminary data showed that the Beijing strain has a high number of clusters in our sample population and this conclusion should be further clarified in the future using the 24 loci MIRU analysis. The high prevalence of Beijing genotype in young age population warrants a close attention to the control policy and the vaccine strategy. These findings indicate that TB is not optimally controlled in Taipei, and that efforts for control strategies should be enforced. Strain analysis, together with virulence studies, will also helping pinpointing isolates associated with higher morbidity and mortality, with the aim of directing efforts to limit the spread of those strains within the region.

## Competing interests

The authors declare that they have no competing interests.

## Authors' contributions

HYD conceived the study, carried out the molecular genetic studies, analyzed the data and drafted the manuscript; IJS participated in the design and carrying out of the survey of anti-tuberculosis drug-resistance, analyzed the data, and provided the clinical isolates for molecular study. WST and SYL carried out mycobacteriological diagnostics, derived clinical isolates, performed identification and drug-susceptibility tests, and provided information about the clinical isolates. JRS and JRC participated in the genotyping studies. CWL and FCT carried out the phylogeny-reconstruction studies, participated in the identification and designation of the SITs, and helped draft the manuscript. JJL conceived the study, participated in its design, helped coordinate the investigation, and helped draft the manuscript. All authors contributed to the study and have read and approved the final manuscript.

## Pre-publication history

The pre-publication history for this paper can be accessed here:



## Supplementary Material

Additional file 1 **MIRU-VNTR patterns of *M. tuberculosis *isolates. Summary of MIRU-VNTR patterns of all MTB isolates.** Click here for file
