# Supplementary material for: Molecular epidemiology and evolutionary genetics of Mycobacterium tuberculosis in Taipei
Source: BMC Infect Dis. 2008 Dec 22;8:170. doi: 10.1186/1471-2334-8-170 (PMC2628671; doi:10.1186/1471-2334-8-170)
Supplement: Additional file 1 — MIRU-VNTR patterns of M. tuberculosis isolates. Summary of MIRU-VNTR patterns of all MTB isolates. [file 1471-2334-8-170-S1.doc]

一、Beijing (187)

| Clade No. No. of strains No. of repeats in MIRU locus^＊^ |
| --- |
| Beijing 1 95 2 2 3 3 2 5 1 7 3 5 3 3  Beijing 2 11 2 2 2 3 2 5 1 7 3 5 3 3  Beijing 3 9 2 2 3 3 2 5 1 6 3 5 3 3  Beijing 4 7 2 2 3 3 2 5 1 6 3 5 4 3  Beijing 5 7 2 2 3 3 2 5 1 8 3 5 3 3  Beijing 6 5 2 2 3 3 2 5 1 7 3 5 2 3  Beijing 7 4 2 2 3 3 2 5 1 4 3 5 3 3  Beijing 8 3 2 2 3 3 2 5 1 6 3 5 4 3  Beijing 9 2 2 2 3 3 2 5 1 7 3 5 3 4  Beijing 10 2 2 2 3 3 2 5 1 5 3 5 1 1  Beijing 11 2 2 2 3 3 2 5 1 5 3 5 3 4  Beijing 12 2 2 2 3 3 2 5 1 7 3 4 3 3  Beijing 13 2 2 2 3 4 2 5 1 7 3 5 3 3  Beijing 14 2 2 2 3 3 2 5 1 7 3 5 3 2  Beijing 15 2 2 2 3 3 2 5 1 7 3 5 3 2  Beijing 16 1 2 1 3 3 2 5 1 7 3 5 3 3  Beijing 17 1 2 2 3 3 2 5 1 7 3 4 3 1  Beijing 18 1 2 2 2 3 2 5 1 7 3 4 3 1  Beijing 19 1 2 2 3 3 2 5 1 5 3 5 3 3  Beijing 20 1 2 2 3 3 2 5 1 6 3 4 3 3  Beijing 21 1 2 0 3 3 2 5 1 1 3 5 3 3  Beijing 22 1 2 2 3 3 2 5 1 10 3 5 2 3  Beijing 23 1 2 2 2 3 2 5 1 7 3 5 3 2  Beijing 24 1 2 2 2 3 2 5 1 7 3 5 3 4  Beijing 25 1 2 2 3 3 2 6 1 7 3 5 4 3  Beijing 26 1 2 2 3 3 2 5 1 7 3 5 1 3  Beijing 27 1 2 2 3 3 2 5 1 7 3 5 2 4  Beijing 28 1 2 2 3 3 2 5 1 5 3 4 3 3  Beijing 29 1 2 2 3 3 2 5 2 7 3 5 3 3  Beijing 30 1 2 2 3 4 2 5 1 6 3 5 3 3  Beijing 31 1 2 2 4 3 2 5 1 6 3 5 3 3  Beijing 32 1 2 1 3 3 2 5 1 9 3 4 3 3  Beijing 33 1 2 2 1 3 1 5 1 7 3 5 3 3  Beijing 34 1 2 2 3 3 2 5 1 6 3 5 3 2  Beijing 35 1 2 2 3 3 2 5 1 7 3 5 3 4  Beijing 36 1 2 2 3 3 2 5 1 5 3 5 3 1  Beijing 37 1 2 2 1 3 2 5 1 7 3 5 3 3  Beijing 38 1 2 2 3 3 2 5 1 6 3 5 4 2  Beijing 39 1 2 2 3 4 2 5 1 10 3 5 3 3  Beijing 40 1 2 2 3 3 2 5 1 7 3 5 4 3  Beijing 41 1 2 2 3 3 2 5 1 10 3 5 2 3  Beijing 42 1 2 2 3 3 2 5 1 7 3 5 3 1  Beijing 43 1 2 2 3 3 2 5 1 6 3 5 3 1  Beijing 44 1 2 2 3 3 2 5 1 6 3 2 3 3  Beijing 45 1 2 2 3 3 2 5 1 7 3 4 3 2  Beijing 46 1 2 1 3 3 2 5 1 7 3 5 3 3  Beijing 47 1 2 2 3 2 2 5 1 7 1 5 3 1 |

| Clade No. No. of different patterns No. of repeats in MIRU locus |
| --- |
| Haarlem 1 10 2 2 2 2 2 5 1 5 3 3 2 3  Haarlem 2 8 2 2 2 3 2 5 1 4 3 3 2 3  Haarlem 3 7 2 2 2 3 2 5 1 5 3 3 2 3  Haarlem 4 2 2 2 2 3 2 5 1 8 3 3 2 3  Haarlem 5 2 2 2 2 2 2 5 1 4 3 3 2 3  Haarlem 6 1 2 2 2 2 2 1 1 1 3 3 2 3  Haarlem 7 1 2 2 2 3 2 5 1 6 3 2 2 3  Haarlem 8 1 2 2 2 3 2 5 1 6 3 3 2 3  Haarlem 9 1 2 2 2 3 2 5 1 5 3 3 2 3  Haarlem 10 1 2 2 2 2 2 5 1 5 2 3 2 1  Haarlem 11 1 2 2 2 3 2 5 1 4 3 3 2 1  Haarlem 12 1 2 2 2 3 2 4 1 5 2 3 2 1  Haarlem 13 1 3 2 2 2 1 5 1 5 3 3 2 1  Haarlem 14 1 2 2 2 3 2 5 1 5 3 3 2 2  Haarlem 15 1 2 2 2 3 2 5 1 4 2 3 2 3  Haarlem 16 1 2 2 2 3 2 5 1 5 2 3 2 3  Haarlem 17 1 1 2 2 3 2 5 1 5 3 3 2 3  Haarlem 18 1 2 2 2 3 2 5 1 6 3 3 1 3  Haarlem 19 1 2 2 2 3 2 5 1 6 2 3 2 3  Haarlem 20 1 2 3' 2 3 2 5 1 5 3 3 2 3  Haarlem 21 1 2 2 2 3 2 3 1 5 3 3 2 3  Haarlem 22 1 2 2 2 3 2 5 1 5 2 2 2 3  Haarlem 23 1 2 2 2 3 2 5 1 5 3 3 2 1  Haarlem 24 1 2 2 2 2 2 5 1 5 2 2 2 3 |

二、Haarlem (48)

三、T family (25)

| Clade No. No. of different patterns No. of repeats in MIRU locus |
| --- |
| T family 1 3 2 4 2 3 2 5 1 5 2 3 2 2  T family 2 3 2 6 2 1 2 5 1 5 2 3 2 2  T family 3 2 2 2 2 3 2 5 1 5 3 3 2 1  T family 4 2 2 3 4 3 2 5 1 5 3 3 2 3  T family 5 1 2 2 5 1 2 5 1 1 3 3 2 3  T family 6 1 2 4 2 3 2 5 1 5 2 2 2 2  T family 7 1 2 2 2 3 2 5 1 4 3 3 2 3  T family 8 1 2 2 4 3 2 5 1 5 3 3 2 4  T family 9 1 2 2 2 3 2 5 1 5 3 3 2 4  T family 10 1 2 2 2 3 2 5 1 5 3 3 2 3  T family 11 1 2 2 3 3 2 6 1 5 3 3 1 1  T family 12 1 2 2 2 2 2 5 1 5 2 3 2 2  T family 13 1 2 4 2 3 2 5 1 5 2 3 2 2  T family 14 1 3 4 2 4 2 5 1 3 2 3 2 4  T family 15 1 2 4 2 3 2 5 1 5 2 3 2 3  T family 16 1 2 4 2 3 2 5 1 4 2 3 2 2  T family 17 1 2 2 2 3 2 5 1 5 3 3 2 1  T family 18 1 2 4 2 2 2 5 1 5 2 3 2 2  T family 19 1 2 1 2 2 2 5 1 1 3 3 2 2 |

四、EIA family (40)

| Clade No. No. of different patterns No. of repeats in MIRU locus |
| --- |
| EIA family 1 28 2 5 4 3 2 6 2 2 3 4 3 2  EIA family 2 6 2 5 4 3 2 6 2 2 3 4 2 2  EIA family 3 2 2 5 4 3 2 6 2 2 3 4 3 4  EIA family 4 1 2 5 4 3 1 6 2 2 3 4 3 2  EIA family 5 1 2 6 4 3 2 6 2 2 3 4 2 2  EIA family 6 1 2 7 4 3 2 6 2 2 3 4 3 4  EIA family 7 1 4 9 4 2 2 5 2 2 3 5 3 3 |

五、U family (8)

| Clade No. No. of different patterns No. of repeats in MIRU locus |
| --- |
| U family 1 2 2 2 8 2 1 5 1 3 3 4 3 3  U family 2 2 2 2 8 2 1 5 1 7 3 4 3 3  U family 3 1 2 5 4 3 2 6 2 2 3 4 3 2  U family 4 1 2 2 8 2 1 5 1 7 3 3 3 3  U family 5 1 2 2 8 2 1 5 1 8 3 3 3 3  U family 6 1 2 2 9 2 2 5 1 4 3 4 3 3 |

六、LAM family (4)

| Clade No. No. of different patterns No. of repeats in MIRU locus |
| --- |
| LAM 1 2 1 2 4 3 2 6 1 5 3 2 2 6  LAM 2 1 1 2 4 3 2 6 1 4 4 2 2 6  LAM 3 1 2 3 3 3 2 6 1 3 3 3 2 3 |

七、MANU2 family (3)

| Clade No. No. of different patterns No. of repeats in MIRU locus |
| --- |
| MANU2 family 1 1 2 4 2 2 2 4 1 6 2 3 2 2  MANU2 family 2 1 1 2 2 3 2 2 1 5 3 2 2 3  MANU2 family 3 1 2 2 3 1 2 5 1 1 2 2 2 2 |

八、BCG (1)

| Clade No. No. of different patterns No. of repeats in MIRU locus |
| --- |
| BCG 1 2 3 2 3 2 4 2 5 3 3 2 2 |

九、Unclassified (40)

| Clade No. No. of different patterns No. of repeats in MIRU locus |
| --- |
| Unclassified 1 4 2 2 2 2 5 1 4 3 3 2 3  Unclassified 2 4 2 2 3 2 5 1 5 3 3 2 3  Unclassified 3 2 4 2 3 2 5 1 5 2 3 2 2  Unclassified 4 2 2 2 2 2 5 1 5 3 3 2 3  Unclassified 5 2 2 2 3 2 5 1 5 3 3 2 1  Unclassified 6 2 2 2 3 2 2 1 5 3 3 2 3  Unclassified 7 1 5 2 3 2 5 1 5 2 3 2 2  Unclassified 8 1 3 2 3 2 5 1 5 2 3 2 2  Unclassified 9 1 2 2 2 2 5 1 5 0 2 2 2  Unclassified 10 1 2 2 2 2 5 1 4 3 2 2 3  Unclassified 11 1 2 2 3 2 5 1 4 3 3 2 3  Unclassified 12 1 2 2 3 2 5 1 4 3 3 2 3  Unclassified 13 1 2 2 3 2 5 1 5 4 3 2 3  Unclassified 14 1 2 2 3 2 5 1 5 3 3 2 3  Unclassified 15 1 2 2 3 2 5 1 5 3 3 1 3  Unclassified 16 1 2 2 3 2 5 1 5 2 3 2 3  Unclassified 17 1 2 2 3 2 5 1 4 2 3 2 3  Unclassified 18 1 1 2 3 2 5 1 4 3 3 2 4  Unclassified 19 1 1 2 4 2 5 1 5 2 3 2 1  Unclassified 20 1 2 7 2 2 5 1 5 2 3 2 1  Unclassified 21 1 2 6 2 2 5 1 7 3 4 3 3  Unclassified 22 1 2 5 2 2 5 1 7 3 4 3 3  Unclassified 23 1 2 8 2 2 5 1 7 3 4 2 3  Unclassified 24 1 2 5 2 2 5 1 4 3 3 3 3  Unclassified 25 1 2 2 3 2 2 1 5 3 2 3 3  Unclassified 26 1 2 4 3 2 2 1 5 3 3 2 4  Unclassified 27 1 10 2 3 2 5 1 5 3 3 2 4  Unclassified 28 1 10 2 N 2 5 1 5 3 3 2 4  Unclassified 29 1 5 4 3 2 6 2 2 3 4 3 2  Unclassified 30 1 5 4 3 1 6 2 2 3 4 3 2 |

^＊^ number of repeats per locus in 12 loci” MIRU2, MIRU4, MIRU10, MIRU 16, MIRU 20, MIRU 23, MIRU 24, MIRU 26, MIRU 27, MIRU 31, MIRU 39, MIRU 40.
